# Vision-Related Quality of Life in Herpetic Anterior Uveitis Patients

**DOI:** 10.1371/journal.pone.0085224

**Published:** 2014-01-02

**Authors:** Lisette Hoeksema, Leonoor I. Los

**Affiliations:** 1 Department of Ophthalmology, University Medical Center Groningen, University of Groningen, Groningen, the Netherlands; 2 W.J. Kolff Institute, Graduate School of Medical Sciences, University of Groningen, Groningen, the Netherlands; University of Illinois at Chicago, United States of America

## Abstract

We investigated the vision-related quality of life (VR-QOL) and the prevalence and severity of depression in patients with herpetic anterior uveitis (AU). This study was conducted in 2012 at the ophthalmology department of the University Medical Center of Groningen (tertiary referral center). We selected patients from an existing uveitis database, all eligible patients were approached. Thirty-six of 66 (55%) patients with herpetic AU (herpes simplex virus or varicella zoster virus) participated, patients were 18 years or older. The diagnosis was made by clinical presentation or a positive anterior chamber tap. All patients received an information letter, informed consent form, National Eye Institute Visual Functioning Questionnaire-25 (NEI VFQ-25), Beck Depression Inventory (BDI-II), Social Support List – Interactions (SSL-I), Social Support List – Discrepancies (SSL-D) and an additional questionnaire for gathering general information. Medical records were reviewed for clinical characteristics. Analyses were conducted on various patient and ocular characteristics. We compared our NEI VFQ-25 scores with those previously found in the literature. Our main outcome measures were VR-QOL, prevalence and severity of depression, social support and various patient and ocular characteristics that could influence the VR-QOL. We found that the NEI VFQ-25 mean overall composite score (OCS) was 88.1±10.6. Compared with other ocular diseases our OCS is relatively high, but lower than that found in a normal working population. The mean general health score was 59.0±19.0; this score is lower than in patients with other ocular diseases, except for untreated Behçet’s patients. Depression was scarce, with only one patient (2.8%) having a moderate depression (BDI-II score of 21). We concluded that herpetic AU affects the VR-QOL in a moderate way. The prevalence of depression in our group of herpetic AU patients was low and therefore does not seem to indicate a need for specific screening and intervention measures in these patients.

## Introduction

Anterior uveitis (AU) is the predominant form of uveitis and herpetic AU is the most frequently observed form of infectious AU. [1] Characteristics like dermatitis, keratitis, elevated intraocular pressure (IOP) and iris sector atrophy are seen in herpetic AU. [2] Also secondary complications, like glaucoma and cataract are reported. [3] Complications of uveitis can lead to irreversible loss of visual functioning. [4] Previous studies showed that 35% of uveitis patients in the Western society are significantly visually impaired or blind [5].

Uveitis is seen in all age groups, and a substantial proportion of patients is of working age. During active uveitis, the inflammation and its treatment may – temporarily – affect visual functioning in such a way that it interferes with reading, computer work, driving, etc. Some patients may lose their job because of – recurrent – uveitis. Fear of a recurrence may cause increased stress levels, even when the uveitis is quiet. This may result in a decreased vision-related quality of life (VR-QOL) and an increased risk of developing a depression.

The purpose of this study is to evaluate the VR-QOL and the prevalence and severity of depression in a group of patients with a specific type of uveitis, i.e. herpetic AU (including herpes simplex virus (HSV) or varicella zoster virus (VZV) related AU). Previous research on a large group of uveitis patients found that uveitis patients reported a markedly poorer visual functioning and general health status than healthy subjects. [6] That study evaluated a non-homogeneous group of uveitis patients with different causes and manifestations of the disease. Since each cause and manifestation of uveitis may differ with regard to clinical characteristics and residual symptoms, it would also be of interest to examine the VR-QOL in the different uveitis entities separately. This may give valuable information for entity-related counseling of patients and may indicate the entity-related need for developing intervention strategies.

## Methods

### Ethics Statement

The Medical Ethical Committee of the University Medical Center of Groningen ruled that approval was not required for this study. The study was conducted according to the tenets of the Declaration of Helsinki.

### Patients

The patients included in this study were selected from an existing database, containing uveitis patients who had been treated or are currently being treated for uveitis at the ophthalmology department of the University Medical Center of Groningen, which is a tertiary referral center. We included 66 patients with herpetic AU. All patients were 18 years or older. The diagnosis was made by clinical presentation (keratitis - dendritic herpes branch - followed by AU, elevated intraocular pressure at presentation, iris sector atrophy developing over time and/or clear facial varicella zoster infection (ophthalmic nerve) with subsequent kerato-uveitis) or a positive anterior chamber tap for local antibody production or the presence of virus DNA by PCR. Patients with other forms or possible causes of uveitis were excluded.

### Data

All 66 patients received an information letter and an informed consent form by mail. Included in this letter, they received the following questionnaires; the National Eye Institute Visual Functioning Questionnaire-25 (NEI VFQ-25), the Beck Depression Inventory (BDI-II), Social Support List – Interactions (SSL-I), Social Support List – Discrepancies (SSL-D) and an additional questionnaire for gathering general information. The patients were asked to complete the questionnaires at home, to sign the informed consent form and to return them by mail.

For measuring the VR-QOL, we used the validated Dutch version of the NEI VFQ-25. The NEI VFQ-25 has been developed by the National Eye Institute. This validated [7], [8] self-administered questionnaire consists of 25 questions, with a total score and subscores ranging from 0–100. In this questionnaire, the score of 0 corresponds to the lowest and of 100 to the highest VR-QOL. There are 12 subscales, each consisting of one or more questions. These subscales are general health, general vision, ocular pain, near activities, distance activities, vision specific social functioning, vision specific mental health, vision specific role difficulties, vision specific dependency, driving, color vision and peripheral vision.

The BDI-II is a validated [9] self-administered questionnaire consisting of 21 questions on how the patient feels and experiences things. Each question can be answered on a four-point scale ranging from 0 to 3. Subscores are added to create a total score. A total score of 0 to 13 corresponds with no depression, of 14 to 19 with a mild depression, of 20 to 28 with a moderately severe depression and of 29 to 63 with a severe depression. The SSL-I and SSL-D are questionnaires developed and validated by the University of Groningen (RUG). These questionnaires measure (1) social interactions between patients and persons with whom they interact and (2) if the received social support corresponds with the desired social support. They each consist of 34 four-choice questions, resulting in scores ranging from 1–4. A high SSL-I score corresponds with sufficient social support. A high SSL-D score corresponds with a deficiency in desired social support. The maximum score of the SSL-I is 136 and of the SSL-D it is 102.

The following information was gathered by the additional questionnaire: present activity of the uveitis, presence of other chronic diseases or diseases with a large impact (recent or in the past), medication use (ocular and other medication), history of depression and/or treatment, and need for visual revalidation. By reviewing medical records, we gathered the following information: present age, sex, unilateral or bilateral AU, systemic disease, follow-up time (defined as time between the start of the first uveitis episode and the end of the last uveitis episode), total time of active disease, total number of uveitis episodes, remission time (defined as time between the end of the last uveitis episode and the date on the questionnaire), Snellen visual acuity (VA), ocular complications in history (elevated IOP, glaucoma, cataract, secondary cataract, keratitis, dry eyes, cystoid macular edema (CME), papillitis, scleritis and herpes zoster ophthalmicus (HZO)) and presence of active uveitis at the time of completing the questionnaire.

Active uveitis was defined as ≥0.5+ cells in the anterior chamber. [10] Transiently elevated IOP was defined as a measured IOP>20 mmHg without pressure reducing medication. Glaucoma was defined as the presence of visual field defects typical for glaucoma that were reproducible and could not be explained by other pathology, with or without glaucomatous disc abnormalities. Dry eyes were defined as the presence of dry eye symptoms and need for artificial tears.

### Statistics

Data were statistically analyzed using SPSS Statistics 20.0.0.1. For the comparison of continuous variables of two groups, we used the Mann-Whitney U test. For comparison of continuous variables of more than two groups, we used the Kruskal–Wallis one-way analysis and the Mann-Whitney U test for post hoc analysis with a Bonferroni correction, using a critical value of 0.05 divided by the number of tests conducted. Correlations were assessed with the Spearman’s Rank Correlations test. For analyzing, Snellen VA was converted to the logarithm of the minimum angle of resolution (logMAR) equivalent. Statistical significance level was set at 0.05.

## Results

Thirty-six of 66 (55%) patients participated by filling out the questionnaires and returning them by mail. Table 1 summarizes the clinical characteristics of the 27 HSV and nine VZV AU patients. Males were slightly overrepresented in relation to females (58 versus 42%). Mean age of the HSV patients was 55.7±17.5 years and of VZV patients it was 63.7±15.1 years (p = 0.201). Complications most frequently observed (in % of patients) were elevated IOP (69%), keratitis (64%), dry eyes (42%) and cataract (36%). We checked and confirmed that all complications developed after the diagnosis of AU. The mean (± SD) of the NEI VFQ-25, BDI-ll, SSL-I and SSL-D scores are given in Table 1. Only one patient had a moderate depression (BDI-II score of 21) at the time of completing the questionnaires, for which he already received medical treatment.

**Table 1:** Clinical characteristics of herpetic AU patients and overall scores on questionnaires (N and (%) or Mean ± SD (range)).

Number of patients	36
HSV/VZV	27 (75%)/9 (25%)
Female/male	15 (42%)/21 (58%)
Unilateral/bilateral	36 (100%)/0 (0.0%)
Age at completing questionnaire (yrs)	57.7±17.1 (25–88)
Follow-up time (yrs)	8.7±12.4 (0.04–41.3)
Number of uveitis episodes	4.7±5.4 (1–27)
Time of active uveitis (months)	5.1±4.3 (1–17)
Remission time (yrs)	3.6±2.5 (0.02–10.7)
Depression in past^a^	4 (11%)
Complications^b^	
- Elevated IOP	25 (69%)
- Keratitis	23 (64%)
- Dry eyes^c^	15 (42%)
- Cataract	13 (36%)
- HZO	8 (22%)
- Glaucoma	5 (14%)
- Secondary cataract	4 (11%)
- CME	2 (6%)
- Scleritis	0 (0%)
- Papillitis	0 (0%)
NEI VFQ-25 OCS^d^	88.1±10.6 (51.7–97.6)
BDI-II score	3.7±4.5 (0–21)
SSL-I score	74.0±17.8 (34–105)
SSL-D score	45.3±16.1 (34–102)

AU: anterior uveitis, HSV: Herpes Simplex Virus, VZV: Varicella Zoster Virus, IOP: Intraocular Pressure, CME: Cystoid Macular Edema, HZO: Herpes Zoster Ophthalmicus, OCS: Overall Composite Score, BDI: Beck Depression Inventory, SSL-I: Social Support List - Interactions, SSL-D: Social Support List - Discrepancies.

^a^ Diagnosed by a physician and medically treated.

^b^ Developed during follow-up AU.

^c^ Medication needed.

^d^ Average of vision-targeted subscale scores, without general health subscore.

Tables 2 and 3 give information on the mean (± SD) of the overall composite score (OCS) and the subscales of the NEI VFQ-25 in the total group. Also, differences herein related to various patient characteristics and ocular variables are presented. The mean OCS in the total group was 88.1±10.6 and the mean general health score was 59.0±19.0.

**Table 2:** NEI VFQ-25 subscale scores and overall composite score (OCS), Mean ± SD.

		GH	GV	OP	NA	DA	VSSF	VSMH	VSRD	VSD	D	CV	PV	OCS^a^
		(n = 36)	(n = 36)	(n = 36)	(n = 36)	(n = 36)	(n = 35)	(n = 36)	(n = 36)	(n = 36)	(n = 30)	(n = 35)	(n = 34)	(n = 36)
Total group (n = 36)		59.0±19.0	76.1±9.3	73.3±22.8	87.7±16.7	92.6±12.6	97.1±8.1	84.9±14.4	84.0±21.9	97.7±7.1	87.1±16.2	97.1±10.1	91.2±20.3	88.1±10.6
Sex	- Male (n = 21)	61.9±23.2	78.1±8.7	81.5±17.1	87.7±18.0	92.5±11.8	96.3±9.2	85.4±11.4	88.1±12.8	97.6±6.0	88.3±15.4	96.3±12.2	91.3±23.3	89.4±10.3
	- Female (n = 15)	55.0±10.4	73.3±9.6	61.7±25.2	87.8±15.4	92.8±14.0	98.3±6.5	84.2±18.3	78.3±30.1	97.8±8.6	84.6±18.2	98.3±6.5	91.1±15.8	86.2±11.1
		*p = 0.19*	*p = 0.14*	***p = 0.02***	*p = 0.99*	*p = 0.54*	*p = 0.30*	*p = 0.61*	*p = 0.58*	*p = 0.35*	*p = 0.50*	*p = 0.71*	*p = 0.59*	*p = 0.24*
Present age (yrs)	- <45 (n = 8)	56.3±17.7	80.0±0.0	75.0±16.4	96.9±6.2	97.9±3.9	100.0±0.0	88.3±5.2	96.9±5.8	100.0±0.0	95.8±7.0	100.0±0.0	96.4±9.4	93.3±3.1
	- 45–65 (n = 14)	62.5±19.0	75.7±11.6	73.2±28.1	83.3±22.2	89.3±17.7	96.4±10.3	81.3±19.1	82.1±22.8	97.0±7.0	85.8±19.2	94.6±14.5	88.5±30.0	86.2±14.7
	- >65 (n = 14)	57.1±20.6	74.3±9.4	72.3±21.5	86.9±13.0	92.9±8.6	96.4±7.6	86.6±12.5	78.6±24.7	97.0±9.0	84.0±15.7	98.2±6.7	91.1±12.4	87.1±7.9
		*p = 0.68*	*p = 0.35*	*p = 0.93*	*p = 0.15*	*p = 0.39*	*p = 0.44*	*p = 0.83*	*p = 0.04^b^*	*p = 0.40*	*p = 0.25*	*p = 0.53*	*p = 0.51*	*p = 0.24*
HSV/VZV	- HSV (n = 27)	61.1±20.0	77.0±9.1	76.4±22.3	89.2±14.6	93.5±11.4	98.6±5.4	87.3±13.1	87.5±18.0	98.8±5.0	88.7±16.2	98.1±6.8	93.0±13.5	89.8±8.9
	- VZV (n = 9)	52.8±15.0	73.3±10.0	63.9±22.9	83.3±22.4	89.8±16.0	93.1±12.7	77.8±16.6	73.6±29.6	94.4±11.0	80.6±15.5	94.4±16.7	86.1±33.3	83.0±14.0
		*p = 0.23*	*p = 0.31*	*p = 0.12*	*p = 0.59*	*p = 0.44*	*p = 0.06*	***p = 0.03***	*p = 0.07*	*p = 0.06*	*p = 0.14*	*p = 0.70*	*p = 0.98*	*p = 0.09*
Activity uveitis	- Active (n = 4)	56.3±12.5	75.0±10.0	71.9±21.3	75.0±28.9	79.2±21.0	87.5±17.7	76.6±19.3	78.1±18.8	95.8±4.8	75.0±16.7	87.5±25.0	68.8±47.3	79.6±19.6
	- Inactive (n = 32)	59.4±19.8	76.3±9.4	73.4±23.3	89.3±14.5	94.3±10.5	98.4±5.3	85.9±13.7	84.8±22.4	97.9±7.3	88.4±15.6	98.4±6.2	94.2±12.6	89.2±8.9
		*p = 0.70*	*p = 0.82*	*p = 0.78*	*p = 0.22*	*p>0.05*	***p = 0.03***	*p = 0.23*	*p = 0.30*	***p = 0.04***	*p = 0.11*	*p = 0.18*	*p = 0.14*	*p = 0.27*
Uveitis episodes^c^	- 1 episode (n = 11)	61.4±17.2	74.5±12.9	61.4±21.3	86.4±15.5	95.5±7.8	96.6±8.1	85.8±12.8	75.0±28.5	96.2±10.1	88.4±13.8	95.5±10.1	95.0±10.5	86.2±8.7
	- >1 episode (n = 22)	56.8±20.7	77.3±7.0	77.8±22.5	88.6±18.6	90.9±14.8	97.6±8.5	84.7±16.1	89.2±17.8	98.5±5.5	87.3±18.1	97.6±10.9	90.5±24.3	89.2±12.1
		*p = 0.65*	*p = 0.36*	***p = 0.04***	*p = 0.52*	*p = 0.47*	*p = 0.51*	*p = 0.92*	*p = 0.06*	*p = 0.44*	*p = 0.96*	*p = 0.26*	*p = 0.95*	*p = 0.11*

NEI VFQ-25: National Eye Institute Visual Functioning Questionnaire-25, GH: General Health, GV: General Vision, OP: Ocular Pain, NA: Near Activities, DA: Distance Activities, VSSF: Vision Specific Social Functioning, VSMH: Vision Specific Mental Health, VSRD: Vision Specific Role Difficulties, VSD: Vision Specific Dependency, D: Driving, CV: Color Vision, PV: Peripheral Vision, OCS: Overall Composite Score, HSV: Herpes Simplex Virus, VZV: Varicella Zoster Virus. Mean scores ± one standard deviation are given.

^a^ Average of vision-targeted subscale scores, without GH. ^b^ Bonferroni correction, significance level p<0.017. ^c^ Missing data in three patients.

**Table 3:** NEI VFQ-25 subscale scores and overall composite score (OCS), Mean ± SD.

		GH	GV	OP	NA	DA	VSSF	VSMH	VSRD	VSD	D	CV	PV	OCS^a^
		(n = 36)	(n = 36)	(n = 36)	(n = 36)	(n = 36)	(n = 35)	(n = 36)	(n = 36)	(n = 36)	(n = 30)	(n = 35)	(n = 34)	(n = 36)
Dry eyes	- No (n = 21)	58.3±19.9	77.1±9.6	79.2±21.8	89.7±16.9	93.3±12.3	97.5±6.5	86.0±14.6	88.1±18.7	98.4±5.7	89.4±15.7	97.5±7.7	94.7±13.4	90.1±9.9
	- Yes (n = 15)	60.0±18.4	74.7±9.2	65.0±22.3	85.0±16.7	91.7±13.4	96.7±10.0	83.3±14.5	78.3±25.2	96.7±8.8	82.5±16.9	96.7±12.9	86.7±26.5	85.3±11.3
		*p = 0.88*	*p = 0.46*	***p = 0.04***	*p = 0.12*	*p = 0.61*	*p = 0.93*	*p = 0.39*	*p = 0.08*	*p = 0.38*	*p = 0.16*	*p = 0.78*	*p = 0.25*	***p = 0.04***
Elevated IOP	- No (n = 11)	68.2±16.2	80.0±8.9	73.9±21.3	94.7±8.6	98.5±3.4	98.9±3.8	89.8±5.8	92.0±12.8	99.2±2.5	93.8±12.4	97.7±7.5	95.5±10.1	92.1±5.6
	- Yes (n = 25)	55.0±19.1	74.4±9.2	73.0±23.8	84.7±18.6	90.0±14.2	96.4±9.4	82.8±16.6	80.5±24.2	97.0±8.3	84.7±16.9	96.9±11.2	89.1±23.6	86.3±11.8
		*p = 0.05*	*p = 0.11*	*p = 0.99*	*p = 0.11*	***p = 0.04***	*p = 0.52*	*p = 0.40*	*p = 0.10*	*p = 0.55*	*p = 0.09*	*p = 0.97*	*p = 0.55*	*p = 0.12*
Keratitis	- No^b^ (n = 20)	53.8±16.8	75.0±8.9	71.9±19.0	89.2±16.5	92.1±11.9	96.1±9.4	85.0±13.5	81.9±23.8	97.1±7.8	85.3±16.1	97.4±11.5	90.3±24.5	87.4±10.8
	- Yes^c^ (n = 16)	65.6±20.2	77.5±10.0	75.0±27.4	85.9±17.4	93.2±13.7	98.4±6.3	84.8±16.0	86.7±19.6	98.4±6.3	88.9±16.6	96.9±8.5	92.2±15.1	88.9±10.7
		*p = 0.08*	*p = 0.46*	*p = 0.37*	*p = 0.58*	*p = 0.38*	*p = 0.24*	*p = 0.87*	*p = 0.47*	*p = 0.27*	*p = 0.42*	*p = 0.50*	*p = 0.87*	*p = 0.39*
Other disease^d^	- No (n = 17)	61.8±17.9	76.5±7.9	74.3±22.3	88.7±13.5	96.1±6.0	99.2±3.1	85.3±11.5	83.8±24.1	96.1±9.8	91.3±9.1	100.0±0.0	95.0±10.4	89.5±6.7
	- Yes (n = 19)	56.6±20.1	75.8±10.7	72.4±23.8	86.8±19.5	89.5±15.9	95.4±10.4	84.5±17.0	84.2±20.3	99.1±2.6	83.8±19.6	94.7±13.4	88.2±25.5	86.8±13.2
		*p = 0.42*	*p = 0.77*	*p = 0.86*	*p = 0.76*	*p = 0.26*	*p = 0.20*	*p = 0.56*	*p = 0.96*	*p = 0.48*	*p = 0.51*	*p = 0.10*	*p = 0.57*	*p = 0.79*
Visual acuity^e,f^	- <0.5 (n = 10)	67.5±16.9	70.0±10.5	68.8±29.0	75.8±21.0	85.0±20.0	93.8±13.5	75.6±22.9	68.8±32.9	95.8±10.6	80.6±20.8	92.5±16.9	77.5±32.2	80.3±16.4
	- ≥0.5 (n = 22)	54.5±19.9	78.2±8.5	74.4±20.6	90.9±13.1	94.7±7.1	98.2±4.5	88.1±7.9	89.2±13.0	98.1±5.7	89.6±14.2	98.8±5.5	96.3±9.2	90.6±5.4
		*p = 0.082*	***p = 0.027***	*p = 0.649*	***p = 0.021***	*p = 0.230*	*p = 0.552*	*p = 0.174*	*p = 0.053*	*p = 0.607*	*p = 0.256*	*p = 0.174*	***p = 0.031***	*p = 0.149*
Treatment uveitis^g^	- No (n = 16)	62.5±18.3	77.5±10.0	74.2±19.6	91.7±12.5	95.8±7.5	97.7±6.8	87.1±11.3	86.7±26.8	97.4±8.5	91.7±12.7	96.9±8.5	96.9±8.5	90.2±8.5
	- Yes (n = 20)	56.3±19.7	75.0±8.9	72.5±25.5	84.6±19.2	90.0±15.2	96.7±9.2	83.1±16.6	81.9±17.4	97.9±6.0	83.6±18.0	97.4±11.5	86.1±26.0	86.4±12.0
		*p = 0.32*	*p = 0.46*	*p = 0.96*	*p = 0.25*	*p = 0.21*	*p = 0.79*	*p = 0.65*	*p = 0.05*	*p = 0.87*	*p = 0.14*	*p = 0.50*	*p = 0.14*	*p = 0.20*

NEI VFQ-25: National Eye Institute Visual Functioning Questionnaire-25, GH: General Health, GV: General Vision, OP: Ocular Pain, NA: Near Activities, DA: Distance Activities, VSSF: Vision Specific Social Functioning, VSMH: Vision Specific Mental Health, VSRD: Vision Specific Role Difficulties, VSD: Vision Specific Dependency, D: Driving, CV: Color Vision, PV: Peripheral Vision, OCS: Overall Composite Score, IOP: Intraocular Pressure. Mean scores ± one standard deviation are given.

^a^ Average of vision-targeted subscale scores, without GH. ^b^ No keratitis in history or keratitis in history without residuals. ^c^ Keratitis with residuals. ^d^ Medical chronic condition or medical condition with large impact, recent or in the past, except for uveitis. ^e^ At least one eye with Snellen visual acuity <0.5. Measured with Snellen chart within six months before or after completing the NEI VFQ-25. ^f^ Missing data in four patients. ^g^ Treatment of the uveitis and/or complications at the moment of completing the NEI VFQ-25.

Female patients scored significantly lower on ocular pain (indicating that they experienced more pain or discomfort around or in the eye). VZV patients scored lower on all subscales and on the OCS compared to HSV patients, but only the difference in scores on vision specific mental health reached significance. Patients with active uveitis had significantly lower vision specific social functioning and vision specific dependency scores. They also had lower distance activities scores, but this did not reach significance. Patients who experienced just one uveitis episode had significantly lower ocular pain scores (more pain) than patients who experienced multiple uveitis episodes. Dry eye patients scored significantly lower on ocular pain (more pain) and on the OCS. Patients with transiently or persistently elevated IOP had significantly lower distance activities scores. Patients with a Snellen VA of less than 0.5 in at least one eye, scored lower on the OCS and on all subscales, but only the scores on general vision, near activities and peripheral vision reached significance.

Age, keratitis and uveitis treatment showed no significant correlation with any of the NEI VFQ-25 outcomes. Also, patients with or without other chronic diseases (in our group: diabetes, respiratory diseases, hypercholesterolemia, hypertension and back pain) or diseases with a large impact (in our group: multiple brain infarcts, cancer, psoriasis vulgaris, depression, antithrombin III deficiency, polymyalgia-rheumatica, hypothyroidism and cardiovascular diseases (recent or in the past)) did not score significantly different on the NEI VFQ-25 scales, including the general health subscale.

Table 4 shows the results of the Spearman’s Rank Correlations tests between studied variables and NEI VFQ-25 subscale scores and OCS. Age at completing the questionnaire and general vision, near activities and vision specific role difficulties were negatively correlated. LogMAR VA of the uveitic eye was negatively correlated with near activities and peripheral vision. Remission time was positively correlated with peripheral vision and central vision. The BDI score was negatively correlated with general health, vision specific social functioning, vision specific mental health, vision specific role difficulties, vision specific dependency, driving and OCS. There was no significant correlation between the subscale scores and OCS and logMAR VA of the healthy eye, duration of active uveitis, total of uveitis episodes, follow-up time, SSL-I score and SSL-D score. There was no significant correlation between the BDI-II score and the SSL-I score or the SSL-D score.

**Table 4:** Spearman’s Rank Correlations between studied variables and NEI VFQ-25 subscale scores and OCS.

	GH	GV	OP	NA	DA	VSSF	VSMH	VSRD	VSD	D	CV	PV	OCS^a^
Age at completing questionnaire	−0.035	**−0.360**	0.097	**−0.371**	−0.220	−0.192	0.057	**−0.407**	−0.113	−0.325	−0.008	−0.206	−0.267
	p = 0.84	**p = 0.03**	p = 0.57	**p = 0.03**	p = 0.20	p = 0.27	p = 0.74	**p = 0.01**	p = 0.51	p = 0.08	p = 0.96	p = 0.24	p = 0.12
LogMAR VA uveitic eye	0.115	−0.273	−0.099	−**0.352**	−0.201	0.029	−0.206	−0.218	−0.116	−0.085	−0.090	−**0.383**	−0.219
	p = 0.53	p = 0.12	p = 0.59	**p = 0.04**	p = 0.26	p = 0.87	p = 0.25	p = 0.22	p = 0.52	p = 0.67	p = 0.63	**p = 0.03**	p = 0.22
LogMAR VA fellow eye	−0.107	−0.261	0.139	−0.243	−0.003	0.049	−0.138	−0.164	−0.122	−0.079	0.173	0.042	−0.130
	p = 0.56	p = 0.15	p = 0.45	p = 0.18	p = 0.99	p = 0.80	p = 0.45	p = 0.37	p = 0.51	p = 0.70	p = 0.35	p = 0.82	p = 0.48
Number of uveitis episodes	−0.008	0.110	0.170	−0.045	−0.271	−0.005	−0.051	0.185	0.142	0.030	0.084	−0.244	0.098
	p = 0.97	p = 0.54	p = 0.34	p = 0.81	p = 0.13	p = 0.98	p = 0.78	p = 0.30	p = 0.43	p = 0.88	p = 0.65	p = 0.19	p = 0.59
Duration of active uveitis	−0.028	0.099	0.143	0.009	−0.193	0.036	0.107	0.011	0.056	−0.058	0.070	−0.155	0.054
	p = 0.88	p = 0.58	p = 0.43	p = 0.96	p = 0.28	p = 0.85	p = 0.55	p = 0.95	p = 0.76	p = 0.77	p = 0.71	p = 0.41	p = 0.76
Follow-up time	0.115	0.144	0.145	0.013	−0.201	0.086	0.061	0.185	0.123	0.068	0.164	−0.250	0.139
	p = 0.51	p = 0.41	p = 0.41	p = 0.94	p = 0.25	p = 0.63	p = 0.73	p = 0.29	p = 0.48	p = 0.72	p = 0.35	p = 0.16	p = 0.43
Remission time	−0.092	0.061	−0.071	0.294	0.291	0.274	0.092	0.046	0.283	−0.075	**0.387**	**0.429**	0.107
	p = 0.59	p = 0.72	p = 0.68	p = 0.08	p = 0.09	p = 0.11	p = 0.59	p = 0.79	p = 0.09	p = 0.69	**p = 0.02**	**p = 0.01**	p = 0.53
BDI-II score	−**0.433**	−0.255	−0.261	−0.131	−0.134	−**0.485**	−**0.493**	−**0.348**	−**0.414**	−**0.558**	−0.066	−0.175	−**0.456**
	**p = 0.01**	p = 0.15	p = 0.14	p = 0.46	p = 0.45	**p = 0.004**	**p = 0.003**	**p = 0.04**	**p = 0.02**	**p = 0.002**	p = 0.71	p = 0.32	**p = 0.007**
SSL-I score	−0.081	−0.211	0.065	−0.143	0.184	0.091	0.088	0.059	0.105	0.237	0.003	0.179	−0.006
	p = 0.67	p = 0.25	p = 0.73	p = 0.44	p = 0.32	p = 0.63	p = 0.64	p = 0.75	p = 0.57	p = 0.24	p = 0.99	p = 0.34	p = 0.97
SSL-D score	−0.024	0.046	−0.060	0.022	−0.066	−0.143	−0.174	−0.197	0.021	−0.356	−0.018	−0.077	−0.130
	p = 0.90	p = 0.81	p = 0.75	p = 0.91	p = 0.73	p = 0.45	p = 0.35	p = 0.29	p = 0.91	p = 0.07	p = 0.93	p = 0.69	p = 0.49

NEI VFQ-25: National Eye Institute Visual Function Questionnaire-25, GH: General Health, GV: General Vision, OP: Ocular Pain, NA: Near Activities, DA: Distance Activities, VSSF: Vision Specific Social Functioning, VSMH: Vision Specific Mental Health, VSRD: Vision Specific Role Difficulties, VSD: Vision Specific Dependency, D: Driving, CV: Color Vision, PV: Peripheral Vision, OCS: Overall Composite Score, VA: Visual Acuity, BDI: Beck Depression Inventory, SSL-I: Social Support List - Interactions, SSL-D: Social Support List – Discrepancies.

^a^ Average of vision-targeted subscale scores, without GH.

## Discussion

We found that in general NEI VFQ-25 subscale scores and the OCS were reasonably high in herpetic AU patients. We found a mean OCS of 88.1 which means that the majority of patients scored between the best possible (100.0) and the second best score (75.0). General health is the only subscale that is not included in the OCS. The mean general health score was lower than the means of the other subscales, namely 59.0. However, this still means that the majority of patients scored their general health between ‘good’ (50.0) and ‘very good’ (75.0). Depression was scarce in our study group, with only one patient having a moderate depression.

To give an overall idea of the height of the scores in our patient group in relation to those previously found in healthy persons and in patients with ocular disease, we constructed Table 5. Interestingly, Hirneiss et al. who obtained NEI VFQ-25 scores in a normal working population as well as in subpopulations thereof with and without ocular disease, found that general health scores were lower in the subgroup with ocular disease. [11] The general health scores of his total group and subpopulations were higher than in our patient group. Studies on patients with noninfectious ocular inflammatory disease and Birdshot chorioretinopathy showed general health scores comparable with our general health score. [12], [13] Highest general health scores were achieved in patients with acute posterior vitreous detachment. [14] Untreated Behçet’s disease patients had the lowest general health scores, which is not surprising because of the commonly associated systemic manifestations in this entity [15].

**Table 5:** NEI VFQ-25 subscale scores and OCS compared with literature.

			GH	GV	OP	NA	DA	VSSF	VSMH	VSRD	VSD	D	CV	PV	OCS^a^
Study	Mean age ± SD (yrs)	Group composition						Mean (SD)							
Hoeksema	58±17	Herpetic anterior	59.0	76.1	73.3	87.7	92.6	97.1	84.9	84.0	97.7	87.1	97.1	91.2	88.1
n = 36		uveitis	(19.0)	(9.3)	(22.8)	(16.7)	(12.6)	(8.1)	(14.4)	(21.9)	(7.1)	(16.2)	(10.1)	(20.3)	(10.6)
Hirneiss2010 [11]	42±9	Normal working	73.0	78.6	85.4	91.9	91.8	97.9	87.4	92.8	98.4	88.7	97.9	93.3	91.1
n = 619		population - Total group	(18.1)	(15.7)	(16.6)	(13.1)	(11.3)	(9.0)	(10.5)	(13.8)	(5.6)	(10.6)	(9.3)	(15.0)	(7.4)
Hirneiss2010 [11]	42±9	Normal working	79.9	79.0	87.6	92.3	92.1	98.1	87.8	93.4	98.5	88.8	98.0	93.4	91.6
n = 511		population - Without	(17.4)	(15.9)	(15.1)	(13.0)	(11.4)	(8.2)	(10.0)	(13.3)	(5.5)	(10.6)	(8.7)	(14.6)	(7.1)
		ocular disease													
Hirneiss2010 [11]	43	Normal working	68.6	79.1	75.1	90.2	90.6	96.8	85.3	89.7	97.9	88.4	97.3	92.5	88.8
n = 108		population - Only with	(20.7)	(15.9)	(19.2)	(13.6)	(10.7)	(12.3)	(12.5)	(15.6)	(5.8)	(10.4)	(11.6)	(16.7)	(8.3)
		ocular disease													
Qian 2011 [12]	41	Noninfectious ocular	60.3	72.8	73.9	79.2	78.8	89.9	70.8	74.2	84.9	77.4	94.9	81.3	79.7
n = 104		inflammatory disease	–	–	–	–	–	–	–	–	–	–	–	–	–
Sakai 2013 [15]	45±14	Behçet uveitis	31.3	48.0	78.8	53.3	60.6	69.6	43.4	53.2	77.3	58.3	82.5	75.0	63.6
n = 20		untreated	(13.8)	(10.1)	(12.9)	(4.9)	(6.8)	(10.2)	(15.3)	(14.0)	(12.7)	(12.2)	(11.8)	(16.2)	(8.9)
Sakai 2013 [15]	45±14	Behçet uveitis	77.5	82.0	98.1	87.4	85.2	90.0	92.6	92.6	95.4	85.0	92.5	93.8	90.3
n = 20		infliximab^b^	(11.2)	(11.1)	(4.6)	(11.9)	(10.8)	(12.6)	(13.7)	(10.2)	(9.9)	(18.5)	(11.8)	(11.1)	(8.7)
Kuiper 2013 [13]	59.5 (median)	Birdshot chorioretinopathy	61.6	63.8	75.1	68.6	70.3	84.5	71.2	64.5	84.2	66.8	80.2	67.6	71.0
n = 105			–	–	–	–	–	–	–	–	–	–	–	–	–
Cahill 2005 [16]	76.4±5.6	Bilateral severe AMD	–	31.4	81.8	29.4	38.8	58.4	34.1	38.2	42.7	16.1	67.5	66.8	–
n = 70			–	(15.8)	(20.3)	(18.6)	(24.7)	(28.1)	(25.1)	(27.1)	(29.7)	(31.3)	(27.7)	(25.1)	–
Schweitzer 2011 [14]	Males:64.5±6.6	Acute posterior vitreous	80.56	85.77	89.58	89.58	94.43	99.11	91.78	95.68	99.40	87.87	99.11	95.53	93.47
n = 84	Females:62.1±7.6	detachment^c^	(15.95)	(10.94)	(12.85)	(10.89)	(8.27)	(3.43)	(9.75)	(8.62)	(3.01)	(14.56)	(6.07)	(11.09)	(6.20)

NEI VFQ-25: National Eye Institute Visual Function Questionnaire-25. GH: General Health, GV: General Vision, OP: Ocular Pain, NA: Near Activities, DA: Distance Activities, VSSF: Vision Specific Social Functioning, VSMH: Vision Specific Mental Health, VSRD: Vision Specific Role Difficulties, VSD: Vision Specific Dependency, D: Driving, CV: Color Vision, PV: Peripheral Vision, OCS: Overall Composite Score.

^a^ Average of vision-targeted subscale scores, without GH. ^b^ 12 months after receiving infliximab. ^c^ Six week follow-up visit.

The overall composite score (OCS) is low in untreated Behçet’s disease and Birdshot chorioretinopathy. [13], [15] Unfortunately, OCS was not given in the study on bilateral age-related macular degeneration patients, but from the subscale scores, it can be derived that it will have been lowest in this patient group. [16] Schiffman et al. also found a relatively low OCS (±63.0) in a large group of uveitis patients. Their data was presented graphically and therefore it is difficult to derive exact values on NEI VFQ-25 scores. Because of this, we did not include that study in Table 5. [6] In our patient group, OCS was relatively high, but lower than those in the working population and in acute posterior vitreous detachment patients. [11], [14] A possible explanation for the relatively high OCS in our study is that all our patients had a unilateral disease.

Looking at subgroup analyses in our study group (Tables 2, 3 and 4), age at the moment of completing the questionnaires seemed to be of no influence on total NEI VFQ-25 scores. When evaluating correlations between age and the NEI VFQ-25 subscales, it seems that general vision, near vision and performing tasks nearby become more difficult with age. Also, older patients more often indicate that accomplishing things is getting more difficult because of reduced vision and they feel limited because of their vision. Surprisingly, a history of keratitis seemed to have no effect on VR-QOL, whereas we would expect that residuals of keratitis would influence visual functioning and thereby some of the vision related subscales of the NEI VFQ-25. Also, any medical chronic condition or medical condition (other than ophthalmologic) with a large impact seemed to have no influence on the NEI VFQ-25 scores. Our study and studies summarized in Table 5 seem to suggest that ophthalmic disease itself may influence general health scores.

In our study, significantly more pain or discomfort in or around the eye was reported by female patients and patients who experienced only one uveitis episode. Previous clinical and epidemiological studies show that women are at an increased risk of developing chronic pain and some evidence suggests that women may experience more severe pain. Multiple biopsychosocial mechanisms may contribute to these gender differences in experienced pain, including sex hormones, endogenous opioid function, genetic factors, pain coping and catastrophizing and gender roles. [17] A possible explanation for the difference in reported pain between patients with one versus multiple uveitis episodes, could be the fact that herpes viruses are neurotrophic, and can destroy sensible nerve fibers. This is well-known for corneal sensibility [18] but may also apply to other structures within the eye. Presumably, repeated herpes activity will have a cumulative effect. Another possibility is that coping strategies may change with a longer duration of the disease.

VZV patients scored somewhat lower on all subscales and OCS compared with HSV patients, and this was not due to age or VA. Eight out of nine (89%) VZV patients had had HZO, and it is therefore possible that the lower scores are at least partly due to the occurrence of dermatitis or post-herpetic neuralgia in these patients. Lukas et al. showed that herpes zoster, and especially post-herpetic neuralgia, is associated with increased levels of pain that have a significant impact on QOL scores. [19] In our study, dry eye patients had a lower OCS, which was mainly due to more ocular pain. Li et al. also reported that VR-QOL in dry eye patients can be impaired [20].

Patients with a Snellen VA of less than 0.5 in at least one eye, were more likely to have lower VR-QOL scores, compared to patients with a Snellen VA of more than 0.5 at both eyes. Of these, only the scores on the subscales general vision, near activities and peripheral vision reached significance. The VA of the uveitic eye was correlated with near activities and peripheral vision scores (Table 4). The VA in the fellow eye does not seem to have any influence on the VR-QOL. The fact that we did not find major significant differences based on VA, was possibly due to the fact that most patients had a relatively good VA in both eyes.

In our study, we identified only one patient (2.8%) with a moderate depression. By comparison, de Graaf et al. found a 12–month prevalence of any mood disorder (i.e. depression and other mood disorders) of 6.1% in the Netherlands between 1996 and 2009. [21] Qian et al. reported that 28/104 (26.9%) of patients with ocular inflammatory disease screened positive for depression, using the BDI-II questionnaire. These depressed patients scored far lower on the composite VFQ-25 score than non-depressed patients. [12] In our study, patients with a higher BDI-II score were also likely to score lower on the VR-QOL. We found that a higher BDI-II score was negatively correlated with general health, vision specific social functioning, vision specific mental health, vision specific role difficulties, vision specific dependency, driving and OCS. A possible explanation for the higher prevalence of depression in the study of Qian et al., is that they included patients with severe posterior and panuveitis in addition to AU patients. Qian et al. also mention that inadequate emotional support is a predictor of depression. In our study, the amount of social support appeared to have no influence on VR-QOL or depression.

The main shortcoming of our study is its modest sample size. Our sample size is considered adequate for overall analyses [22], but it may be too limited for all subgroup analyses, resulting in an underreporting of possibly relevant associations. Also, only 55% of herpetic uveitis patients participated in the present study, which may have resulted in a selection bias. Furthermore, our patients were seen at a tertiary referral center and therefore this population may not represent the general uveitis population.

In conclusion, herpetic AU affects the VR-QOL, but only in a moderate way. The NEI VFQ-25 subscale scores and OCS are reasonably good. The prevalence of depression in our group of herpetic AU patients was low and therefore does not seem to indicate a need for specific screening and intervention measures in this specific patient group.
